# Determinants of exercise adherence and maintenance among cancer survivors: a systematic review

**DOI:** 10.1186/1479-5868-11-80

**Published:** 2014-07-02

**Authors:** Caroline S Kampshoff, Femke Jansen, Willem van Mechelen, Anne M May, Johannes Brug, Mai JM Chinapaw, Laurien M Buffart

**Affiliations:** 1 Department of Public and Occupational Health, and the EMGO+ Institute for Health and Care Research, VU University Medical Center, Amsterdam, The Netherlands; 2 Department of Otolaryngology/Head & Neck Surgery, VU University Medical Center, Amsterdam, The Netherlands; 3 Julius Center, University Medical Center Utrecht, Utrecht, The Netherlands; 4 Department of Epidemiology and Biostatistics and the EMGO+ Institute for Health and Care Research, VU University Medical Center, Van der Boechorststraat 7, 1081 Amsterdam, The Netherlands

**Keywords:** Physical activity, Exercise, Intervention adherence, Determinants, Neoplasms, Behavior, Systematic review

## Abstract

For an exercise intervention to be successful, it is important that cancer survivors adhere to the prescribed program. To be able to improve adherence and to preserve achieved beneficial effects, insights into the relevant and modifiable determinants is important. Therefore, we aimed to systematically review determinants of exercise adherence and maintenance in cancer survivors using a socio-ecological approach.

Studies were identified in PubMed, Embase, PsycINFO and SPORTDiscus up to July 2013. We included full-text articles that: 1) were conducted among adult cancer survivors; 2) quantitatively assessed factors associated with intervention adherence and maintenance, and 3) were published in English. The methodological quality of the selected studies was examined. A best evidence synthesis was applied.

Eighteen studies were included. Median methodological quality was 53% and ranged from 21-78% of maximum score. Twelve studies focused on determinants of exercise adherence and evaluated 71 potential determinants: 29 demographic and clinical, 27 psychological, ten physical, four social factors, and one environmental factor. Six studies focused on determinants of exercise maintenance after completion of an intervention, and investigated 63 factors: 22 demographic and clinical, 28 psychosocial, nine physical, three social and one environmental factor. We found moderate evidence for a positive association between exercise history and exercise adherence. Inconsistent findings were found for age, gender and education as well as for psychological factors such as stage of change, perceived behavioral control, self-efficacy, extraversion, attitude, intention, fatigue, and quality of life, and physical factors including cardiovascular fitness, body mass index, and baseline physical activity.

Exercise history is positively associated with exercise adherence. Future trials should further study the influence of social and environmental determinants on exercise adherence and maintenance in addition to demographic, psychological and physical determinants.

## Introduction

The 5-year survival rates across all cancers have increased in the United States from 49% in 1975–1977 to 68% in 2003–2009 [1]. In the Netherlands, the 5-year survival rates across all cancers have increased to 54% for men and 62% for women in 2001–2010 [2]. Besides these major advances in disease free and overall survival rates, many cancer survivors face physical and psychological problems such as reduced physical fitness [3] and quality of life [4]. Physical activity (PA i.e., any bodily movement that results in energy expenditure [5]) and exercise (i.e. specific type of PA that is planned, structured, and repetitive and aims to improve or maintain physical fitness, performance or health [5]) are increasingly recognized as promising interventions aiming to counteract cancer- and treatment-related problems [6]. Systematic reviews and meta-analysis showed beneficial effects of exercise programs on aerobic fitness [7], muscle strength [8], quality of life [9-11], fatigue [12] and depression [13], however the reported effect sizes are generally small to moderate, varying from 0.10 to 0.54 [14-16].

International evidence-based physical activity guidelines recommend exercise programs as a conditional part of care for all cancer survivors [16-20]. For an exercise program to be successful, it is important that cancer survivors adhere to the prescribed program. Yet, exercise adherence during and after cancer treatment is reported as challenging [21]. Adherence can be defined as the degree of attendance or completion of prescribed exercise sessions [22]. To be able to improve adherence, insights into its relevant and modifiable determinants is important. Previous reviews showed that cancer survivors’ exercise stage of change, exercise intention and perceived behavioral control were significantly associated with exercise intervention adherence [23,24]. Furthermore, demographic determinants such as lower age and lower body mass index (BMI) were found to be associated with exercise intervention adherence [25].

In order to receive a better understanding of exercise adherence, socio-ecological models of determinants of health behaviors posit that potential social and environmental determinants should be taken into account in addition to demographic, physical, and psychological determinants [26,27]. However, previous reviews on determinants of exercise adherence among cancer survivors lack a complete overview of different types of determinants, a thorough methodological quality assessment, or a presentation of findings from multivariate analysis [23,25]. Furthermore, Courneya and colleagues [28] suggested that determinants of adherence to exercise during cancer treatment may differ from determinants after completion of primary cancer treatment. The Physical Activity and Cancer Control (PACC) framework [6] distinguishes four time periods after a cancer diagnosis: pretreatment, during treatment, survivorship and end-of-life. Little is known about the most important determinants in the different time periods.

To be able to preserve achieved beneficial effects on physical and psychological outcomes, cancer survivors need to maintain exercising after completion of an exercise intervention. Maintaining higher levels of exercise may also reduce the risk of cancer death and recurrence [29-31]. Despite beneficial effects, for many cancer survivors it appears to be difficult maintain sufficient levels of PA [32]. Hence, a better understanding of determinants of exercise maintenance is needed.

In summary, in cancer survivors, little is known about the determinants of exercise adherence and maintenance in the different phases of cancer survivorship. Identifying these determinants provides insight into possible opportunities to optimize adherence to exercise interventions for cancer survivors, and may help health care professionals to personalize future interventions and target specific patient groups who need additional support (e.g. low adherers or maintainers). Therefore, the aim of this systematic literature review is to identify determinants of exercise adherence and exercise maintenance. In addition, we aim to differentiate between determinants of exercise adherence in cancer survivors before, during and after primary cancer treatment according to the PACC framework [33].

## Methods

### Literature search

The databases, PubMed (dates of coverage: 1950-present), Embase (1947-present), PsycINFO (1880-present) and SPORTDiscus (1800-present), were searched from inception to July 2013. An information specialist of the VU University Medical Center was consulted for the development of the search strategy. Relevant keywords included terms related to the intervention (e.g. physical activity, exercise, sports, training) AND the participants (e.g. cancer, neoplasm, tumor) AND adherence (e.g. adherence, adaptation) AND relevant personal and environmental factors (e.g. correlates, determinants). The full search strategy is available on request. In addition, studies were identified from reference lists of relevant studies retrieved from the primary search.

### Eligibility criteria

Studies were included if: 1) they were performed in adult (≥18 years) cancer survivors before, during and/or after primary cancer treatment; 2) they quantitatively assessed factors associated with exercise intervention adherence or factors associated with exercise maintenance after completion of an intervention; 3) original full-text was available in English. Studies were excluded if they reported on an exercise intervention consisting of a PA recommendation only, factors associated with adherence to a lifestyle intervention that combined exercise with other behaviors (e.g. diet) or a yoga intervention consisting of breathing techniques, relaxation or meditation only.

### Selection process and data extraction

Screening of all four databases was performed in two phases. First, titles and abstracts of identified articles were screened by two independent reviewers (CK and FJ) to exclude articles out of scope. In case of disagreement, the full-text was screened for eligibility. Second, full-texts of the retrieved articles were screened for eligibility by both reviewers. Disagreement between the two reviewers was resolved by discussion. When necessary, a third reviewer (LB) was consulted.

Next, data was extracted using a standardized form including the following items: cancer diagnosis, study population (including the number, age and gender of patients), type of exercise intervention, cancer-related time period, adherence or maintenance rates and definitions, and results (i.e. potential determinants of exercise adherence or maintenance). Determinants of exercise adherence and exercise maintenance were assessed separately. Each factor was scored as positively (+) or negatively associated (−) if the association was statistically significant (p < 0.05), or borderline significant (p < 0.10), otherwise, we labeled the factor as ‘no evidence for an association’ (0). In case included studies evaluated the associations using both univariate and multivariate analyses, we used the results from the multivariate analysis.

### Categorization of determinants

Determinants were categorized into five groups according to the ecological model of health behavior; (i) demographic and clinical (e.g. age, stage of disease and date of diagnosis), (ii) psychological (e.g. Trans Theoretical Model (TTM) stage of change and health-related quality of life), (iii) physical (e.g. past exercise behavior, muscle strength and body composition), (iv) social (e.g. family support), and (v) environmental factors (e.g. location of fitness center). Determinants of exercise adherence were categorized into three time periods after cancer diagnosis according to the PACC framework: pretreatment, during treatment and after treatment (survivorship and end-of-life care).

### Methodological quality assessment

The methodological quality of the included studies was assessed using an 11-item methodological quality assessment tool adapted from existing quality criteria lists [34-36]. The quality list included items on (i) study population and participation (three items); (ii) study attrition (two items); (iii) data collection (three items) and (iv) data analysis (three items) (Table 1). Further, the items distinguished between informativeness (I, three items) and validity/precision (V/P, eight items) [34].

**Table 1:** Methodological quality assessment tool and quality score of the included studies (n = 18)

		**Exercise intervention adherence**												**Exercise maintenance after completion of an intervention**						
		**Pre**	**During**				**After**					**During/after**								
**Items/reference**		[39]	[40]	[42]	[43]	[41]	[45]	[47]	[44]	[46]	[48]	[49]	[50]	[56]	[53]	[52]	[51]	[55]	[54]	**Score (%)**
**Study population and participation**	Topic																			
A. Description of cancer type, stage and treatment	I	1	1	1	1	1	1	1	1	1	1	1	1	1	1	1	1	1	1	100
B. Description of inclusion and exclusion criteria	I	1	1	1	1	1	1	1	1	1	1	1	1	1	1	1	1	1	1	100
C. Positive if the participation rate at baseline was at least 80%, or if the non-response was not selective^a^	V/P	0	0	0	0	0	0	0	0	0	0	1	0	?	0	0	1	0	0	16
**Study attrition**																				
D. Number of patients included in the analysis ≥100	V	0	1	0	0	0	0	1	0	0	0	0	0	1	1	1	0	0	0	26
E. Positive if the response at first follow-up was at least 80%, or if the non-response at first follow-up was not selective^b^	V/P	0	1	1	1	0	1	1	1	1	1	1	1	1	1	1	1	1	0	80
**Data collection**																				
F. Positive if determinants of adherence were measured with a reliable tool^c^	V/P	0.5	0.7	0.5	0.6	0.6	0.8	0.6	0.8	0.7	0.7	0.5	0.8	0.7	0.8	0.7	0.4	0.5	0.7	63
G. Positive if determinants of adherence were measured with a valid tool^d^	V/P	0.2	0.5	0.5	0.2	0.6	0.5	0.6	0.8	0.6	0.6	0.2	0.6	0.7	0.6	0.4	0	0	0	40
																				
H. Adherence was measured by an objective tool^e^	V/P	1	1	0	1	1	1	0	0	0	0	0	1	0	0	0	0	1	0	37
**Data analysis**																				
I. Multivariate analysis techniques was used.	V/P	0	1	1	1	0	1	1	1	1	0	1	1	1	1	1	0	0	1	38
J. Results were presented as point estimates (mean differences/Beta’s/correlation coefficients) and measures of variability (SD, standard error or CI)	I	1	0	0	0	0	0	0	0	1	1	0	0	0	0	1	1	1	1	37
K. Positive if number of samples is at least 10 times the number of independent variables	V/P	0	1	1	0	0	1	1	1	0	0	1	0	1	1	0	0	0	0	42
**Total quality score (%)**^f^		**22**	**78**	**51**	**47**	**28**	**67**	**64**	**57**	**54**	**29**	**59**	**55**	**68**	**55**	**52**	**43**	**31**	**21**	

1, study provided information on the quality item and met the criterion; 0, study provided information on the quality item but did not meet the criterion; ?,study provided no or insufficient information on the quality item.

I: informativeness; V: validity/P: precision. ^a^attrition analyses were performed and results showed no significant differences between baseline study sample and population of eligible subjects; ^b^attrition analyses were performed and results showed no significant differences between dropouts and follow‒up participants; ^c^associated factors showed internal consistency of Cronbach's alpha ≥0.70 or test‒retest correlations of ≥0.80 or κ/ICC ≥0.70. For clinical factors a standardized protocol was followed by trained researchers; ^d^associated factors showed correlations of ≥0.80 or κ/ICC ≥0.70 with similar constructs. For physical variables (i.e., past physical activity and past sedentary behavior) an objective measurement instrument (i.e., accelerometer/pedometer) was used. For clinical variables a standardized protocol was followed by trained researchers; ^e^For walking interventions: adherence or maintenance was measured by accelerometer or pedometer read out by the researcher. For supervised exercise : the trainer reported presence of the participant; ^f^the number of items scored positively on the validity/precision (V/P) criteria divided by the total number of validity/precision criteria (i.e. 8).

Two reviewers (CK and FJ) independently conducted the quality assessment. If the study provided information on a quality item and met the criterion, we gave a positive score. If the study provided information on a quality item but did not meet the criterion, we gave a negative score. In case of no or insufficient information, we scored the quality item with a question mark. When an article referred to another study containing relevant information for scoring the quality items, the study of interest was retrieved. If the additional study did not provide the requested information, a question mark was given. For items on reliability and validity of a measurement tool (items F and G), we separately evaluated the reliability and validity of the measurement tool used for each individual factor, and weighed the scores. For example, if a study assessed 20 singular associated factors of which 11 were measured with a reliable tool, a score of 0.55 (11/20) was given for reliability. Therefore, the total score for item F and G ranged from 0 to 1.

Disagreements in the methodological quality assessment were resolved by discussion and, if necessary, by consulting the third reviewer (LB). For each study, we calculated a total methodological quality score by counting the number of items scored positively on the validity/precision (V/P) criteria divided by the total number of validity/precision criteria (i.e. 8). According to Chinapaw and colleagues [34] the three informativeness (I) criteria were omitted from our calculation, because these criteria represent descriptive information only. Therefore, the total score of methodological quality could range from 0 to 8. We defined a study to be of ‘high methodological quality’ when it scored ≥70% of the criteria as positive (+) and of ‘low methodological quality’ when it scored <70% of the criteria as positive [37].

### Level of evidence

To synthesize the methodological quality of the studies and to be able to draw conclusions regarding the determinants of exercise adherence and maintenance, we applied a best-evidence synthesis. This rating system consists of three levels and takes into account the number, methodological quality and consistency of outcomes of the studies as follows [37,38]: A) strong evidence: consistent findings in multiple (≥2) high-quality studies; B) moderate evidence: consistent findings in one high quality study and at least one low-quality study, or consistent findings in multiple (≥2) low-quality studies; C) insufficient evidence: only one study available or inconsistent findings in multiple (≥2) studies. Results were considered to be consistent when at least 75% of the studies showed results in the same direction.

## Results

**Figure 1:**
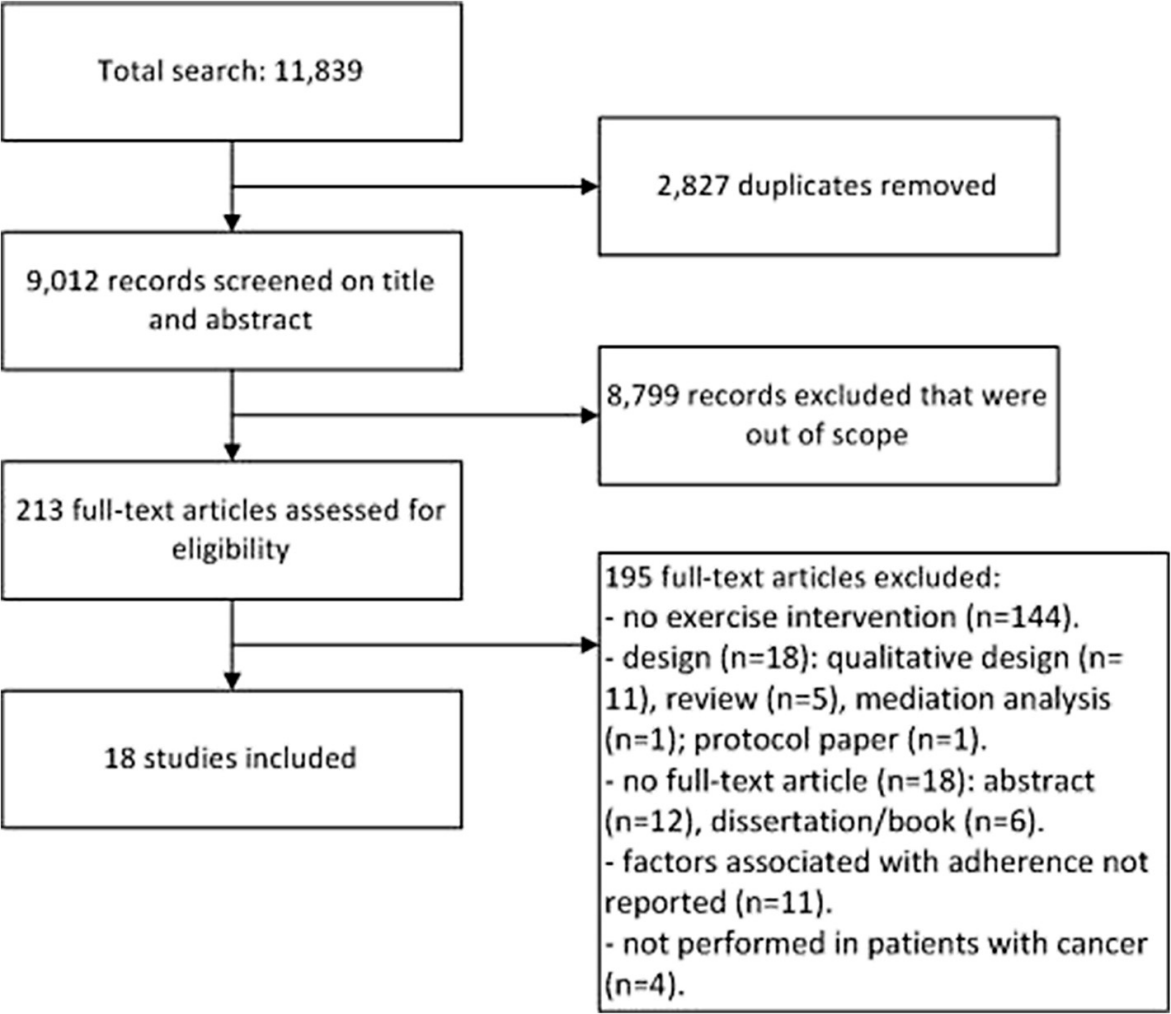
Flowchart of conducted literature search and study inclusion.

The electronically database search yielded 11,839 records. After removing duplicates, 9,012 titles and abstracts were screened and 213 potentially relevant articles were retrieved in full-text. Finally, 18 articles met the in- and exclusion of the present review (Figure 1).

Main study characteristics, including the type of cancer, study population, exercise intervention, and definition and results of exercise adherence or maintenance are presented in Table 2. One study focused on determinants of exercise intervention adherence before treatment [39], four studies during treatment [40-43], five studies after treatment [44-48], and two studies during and after treatment [49,50]. Six studies focused on determinants of exercise maintenance [51-56]. Three studies examined determinants of exercise adherence [40,44,49] and maintenance [51-53] in the same sample, but published in separate articles.

**Table 2:** Characteristics of the included studies (n = 18)

**First author, year**	**Cancer diagnosis**	**Study population (number of patients (n); mean age ± SD; %female)**	**Study design**	**Exercise intervention**	**Outcome measures adherence or maintenance**	**Adherence/maintenance (% or mean ± SD)**
**Pretreatment**						
Peddle, 2009 [39]	Lung cancer	n = 19; 64 ± 10y; 68% female	Single-group trial	AET 5 times a week for the duration of surgical wait time (range 4–13 weeks)	Percentage of the prescribed number of sessions attended	73 ± 35%
**During treatment**						
Courneya, 2008 [40]	Breast cancer	n = 160; 49y; 100% female	Three-armed RCT	1) Supervised AET or 2) RET, 3 times a week for the duration of CT (ranging from 12 to 24 weeks)	Percentage of the expected number of sessions attended	Group 1: 72.0 ± 30.1%; Group 2: 68.2 ± 28.4%
Klepin, 2011 [41]	Acute myelogenous leukemia	n = 24; 65.1 ± 7.8y; 62.5% female	Single-group trial	Supervised AET and strength training 3 times a week for 4 weeks	Number of exercise sessions completed	2.7 ± 2.4
Shang, 2012 [42]	Mixed (34% breast cancer)	N = 68; 59.8 ± 10.8y; 39.7% female	Two-armed RCT	Home-based walking intervention 5 times a week for the duration of RT/CT (ranging from 5 to 35 weeks)	Percentage of patients meeting the personalized exercise prescription > 2/3 of the study period	67.7%
Swenson, 2010 [43]	Breast cancer	n = 29; 46.9y (range: 40–54); 100% female	Two-armed RCT	Tools and advise to perform minimal 10,000 steps per day (PA assessed over 12 months)	Percentage of patients meeting the exercise prescription of 10,000 steps per day	74%
**After treatment**						
Courneya, 2004 [44]	Colorectal cancer	n = 62; 59.9 ± 10.7y; 45.2% female	Two-armed RCT	Home-based AET 3–5 times a week for 16 weeks	Average min/week of moderate-strenuous AET performed	91.5 ± 148.4 min/week
Courneya, 2004 [45]	Prostate cancer	n = 82; 68.2 ± 7.9y; 0% female	Two-armed RCT	RET at fitness center 3 times a week for 12 weeks	Number of observed exercise session attended	28.2 ± 7.1
Latka, 2009 [46]	Breast cancer	n = 37; 56.5 ± 9.5y; 100% female	Two-armed RCT	Supervised AET 3 times a week and home-based AET 2 times a week for 6 months	Average min/week of moderate-intensity AET performed (prescribed 150 min.)	122.8 ± 52.4 min/week
McGuire, 2011 [47]	Breast cancer	n = 120; 58.7y, 100% female	Two-armed RCT	Home-based strength training for 8 months and strength training in a fitness center for the following 16 months, both 2 times a week	Percentage of the prescribed number of sessions performed	62%
Pinto, 2009 [48]	Breast cancer	n = 43; 53.4 ± 9.1; 100% female	Two-armed RCT	Home-based walking intervention 2–5 days a week for 12 weeks	Percentage of patients meeting the exercise prescription	54-91%
**During and after treatment**						
Courneya, 2002 [49]	Mixed (41% breast cancer)	n = 51; 52.5 ± 10.2y; 84.4% female	Two-armed RCT	Home-based AET 3–5 times per week for 10 weeks	Average min/week of moderate-strenuous AET performed	141.1 ± 129.2 min/week
Courneya, 2010 [50]	Lymphoma	n = 60; 52.8y (range: 18–77); 38.3% female	Two-armed RCT	Supervised AET 3 times a week for 12 weeks	Percentage of the expected number of sessions attended	78 ± 30%
**Maintenance**						
Courneya, 2004 [51]	Mixed (40% breast cancer)	n = 30; 54.9 ± 8.0y; 77% female	Two-armed RCT	Home-based AET 3–5 times per week for 10 weeks	Average min/week of moderate-strenuous exercise	146.3 ± 143.5 min/week
Courneya, 2009 [52]	Breast cancer	n = 201; 49y; 100% female	Three-armed RCT	1) Supervised AET; 2) RET 3 times a week for the duration of CT (ranging from 12 to 24 weeks) or 3) a delayed 1 month supervised program for usual care patients	Meeting AET and/or RET guidelines	Neither: 42.3%; either: 36.8%; both: 20.9%
Courneya, 2011 [53]	Lymphoma	n = 110; 44 pt <55y and 66 pt ≥55y; 43.6% female	Two-armed RCT	1) Supervised AET 3 times a week for 12 weeks or 2) a delayed 1 month supervised program for usual care	Percentage of patients meeting ACSM guideline	55.5%
Loprinzi, 2012 [54]	Breast cancer	n = 69; 70.6 ± 1.2y; 100% female	Three-armed RCT	1) Supervised AET; 2) supervised RET or 3) supervised stretching and relaxation exercise 3 times a week for 12 months	Activity status based on TTM stages	Sufficiently active: 57%; Insufficiently active: 43%
Rogers, 2011 [55]	Breast cancer	n = 36; 53 ± 9y; 100% female	Two-armed RCT	1) 12 individual supervised exercise sessions, 6 discussion group sessions and 3 individual face-to-face counseling sessions over a 3 month period or 2) information on PA after a cancer diagnosis	Daily minutes of activity of moderate-strenuous activity	Group 1: 174.9 min/day; Group 2: 92 min/day
Vallance, 2010 [56]	Breast cancer	n = 266; 57y (range 36–90); 100% female	Four-armed RCT	Exercise recommendation and 1) nothing, 2) exercise for health book, 3) pedometer or 4)exercise for health book and pedometer	Percentage of patients meeting ACSM guideline	49.2%

ACSM = American College of Sports Medicine; AET = aerobic exercise training; CT = chemotherapy; PA = physical activity; RET = resistance exercise training; RT = radiotherapy; TTM = Transtheoretical Model.

### Outcome measures of adherence and maintenance

In four studies [39,40,47,50], exercise adherence was defined as a percentage of the prescribed number of sessions attended (mean: 62-78%), three studies [44,46,49] used the average minutes of exercise per week (mean: 92–141 min/week), three studies [42,43,48] used the percentage of survivors meeting the exercise prescriptions (mean: 54-74%), and two studies [41,45] used the number of completed exercise sessions (mean: 3 of 12 [41] and 28 of 36 [45] sessions), see Table 2. Maintenance was defined by average minutes of PA per week (mean: 92–175) in two studies [51,55], by percentage of survivors meeting the PA guideline (mean: 37-56%) in three studies [52,53,56], and the number of survivors in the action or maintenance stage of change compared to the number of survivors in the precontemplation, contemplation or preparation stage (i.e. 57% and 43%) in one study [54].

### Methodological quality

The median methodological quality score of the included studies was 53% and the range was 21% to 78% (Table 1). One study [40] was of high methodological quality. Of all studies, 84% had shortcomings related to the selection of the study sample (item C), 74% had shortcomings related to the sample size (item D), and 63% had shortcomings related to the assessment of adherence (item H), and analysis (item J).

### Determinants of exercise adherence

Twelve studies focused on determinants of exercise adherence and evaluated 71 factors: 29 demographic and clinical, 27 psychological, ten physical, four social factors, and one environmental factor. In total, 19 demographic and clinical, 18 psychological, and eight physical factors, and one environmental factor were examined in two or more studies (Table 3).

**Table 3:** Determinants of exercise adherence

	**Overall**					**During treatment**				**After treatment**			
	**N**	**N + (ref)**	**N- (ref)**	**N0 (ref)**	**LoE**	**N**	**N + (ref)**	**N- (ref)**	**N0 (ref)**	**N**	**N + (ref)**	**N- (ref)**	**N0 (ref)**
**Demographic & Clinical**													
Age	12	1 [50]	1 [45]	10 [39-49]	C	4			4 [40-43]	5		1 [45]	4 [44,46-48]
Being married	10	1 [47]	1 [42]	8 [40,41,44-46,48-50]	C	3		1 [42]	2 [40,41]	5	1 [47]		4 [44-46,48]
Education	9			9 [40-42,44-46,48-50]	C	3			3 [40-42]	4			4 [44-46,48]
Employment	8		1 [44]	7 [40,42,43,45,47,49,50]	C	3			3 [40,42,43]	3		1 [44]	2 [45,47]
Gender	6	2 [39,49]		4 [41,42,44,50]	C	2			2 [41,42]	1			1 [44]
Income	5			5 [40,44,45,49,50]	C	1			1 [40]	2			2 [44,45]
Smoking	4		1 [50]	3 [39,40,45]	C	1			1 [40]	1			1 [45]
Race	2			2 [41,42]	C	2			2 [41,42]	0			
Disease stage	10	1 [40]		9 [42-50]	C	3	1 [40]		2 [42,43]	5			5 [44-48]
Time since diagnosis/treatment	7			7 [44-50]	C	0				5			5 [44-48]
Type of treatment	5	2 [44,50]		3 [42,46,47]	C	1			1 [42]	3	1 [44]		2 [46,47]
Tumor localization	4			4 [42,44,49,50]	C	1			1 [42]	1			1 [44]
Type of surgery	4			4 [40,43,44,46]	C	2			2 [40,43]	2			2 [44,46]
Radiotherapy	4			4 [43-45,49]	C	1			1 [43]	2			2 [44,45]
Chemotherapy	3			3 [44,49,50]	C	0				1			1 [44]
Comorbidity	3			3 [39,41,47]	C	1			1 [41]	1			1 [47]
Chemotherapy cycle	2			2 [43,50]	C	1			1 [43]	0			
Type of chemotherapy	2			2 [40,50]	C	1			1 [40]	0			
Surgery	2			2 [45,49]	C	0				1			1 [45]
**Psychological**													
Attitude	6			6 [39,40,44,45,49,50]	C	1			1 [40]	2			2 [44,45]
Intention	6	1 [45]		5 [39,40,44,49,50]	C	1			1 [40]	2	1 [45]		1 [44]
Perceived behavioral control	6	3 [39,44,49]		3 [40,45,50]	C	1			1 [40]	2	1 [44]		1 [45]
Social norms	6			6 [39,40,44,45,49,50]	C	1			1 [40]	2			2 [44,45]
Quality of life	6			6 [40,41,43,45,46,50]	C	3			3 [40,41,43]	2			2 [45,46]
Stage of change	5	3 [44-46]		2 [45,48]	C	0				5	3 [44-46]		2 [45,48]
Fatigue	5		1 [42]	4 [40,43,45,50]	C	3		1 [42]	2 [40,43]	1			1 [45]
Depression	4		1 [40]	3 [41,46,50]	C	2		1 [40]	1 [41]	1			1 [46]
Self-efficacy	3	1 [48]		2 [39,50]	C	0				1	1 [48]		
Anxiety	3			3 [40,46,50]	C	1			1 [40]	1			1 [46]
Extraversion	2	1 [49]		1 [44]	C	0				1			1 [44]
Distress	2			2 [41,42]	C	2			2 [41,42]	0			
Neuroticism	2			2 [44,49]	C	0				1			1 [44]
Openness	2			2 [44,49]	C	0				1			1 [44]
Agreeable	2			2 [44,49]	C	0				1			1 [44]
Conscientiousness	2			2 [44,49]	C	0				1			1 [44]
Self-esteem	2			2 [40,50]	C	1			1 [40]	0			
Happiness	2			2 [46,50]	C	0				1			1 [46]
**Physical**													
Exercise history	3	3 [42,47,50]			B	1	1 [42]			1	1 [47,50]		
Body mass index	10		2 [46,50]	8 [39-45,47]	C	4			4 [40-43]	4		1 [46]	3 [44,45,47]
PA at baseline	7			7 [40,41,43,44,46,48,49]	C	3			3 [40,41,43]	3			3 [44,46,48]
Body composition	6			6 [40,43,44,46,49,50]	C	2			2 [40,43]	2			2 [44,46]
Cardiovascular fitness	5	2 [40,42]		3 [44,49,50]	C	2	2 [40,42]			1			1 [44]
Physical functioning	4	1 [41]		3 [42,43,50]	C	3	1 [41]		2 [42,43]	0			
Muscle strength	3			3 [40,41,45]	C	2			2 [40,41]	1			1 [45]
Flexibility	2			2 [44,49]	C	0				1			1 [44]
**Environmental**													
Fitness center	2	1 [40]		1 [45]	C	1	1 [40]			1			1 [45]

N+, number of studies showing a positive association; N-, number of studies showing a negative association; N0, number of studies showing no association; LoE, Level of Evidence: A. strong evidence; B. moderate evidence; C. insufficient evidence.

PA: physical activity.

We found moderate evidence that exercise history was positively associated with exercise adherence during and after cancer treatment (Table 3). Due to inconsistent findings, there was insufficient evidence for an association of gender, type of treatment, perceived behavioral control, stage of change, self-efficacy, extraversion, cardiovascular fitness, and fitness center with exercise adherence. Education level, income, time since diagnosis or treatment, tumor localization, type of surgery, radiotherapy, chemotherapy, comorbidity, attitude, social norms, quality of life, anxiety, baseline PA, body composition and muscle strength were examined in three or more studies, which all found no significant association with exercise adherence (Table 3). Insufficient evidence was also found for ten demographic and clinical, nine psychological, two physical, and four social factors that were studied in one single article (Table 4).

**Table 4:** Determinants of exercise adherence or maintenance examined in one single study (insufficient evidence)

**Demographic & Clinical**		**Psychological**		**Physical**		**Social**		**Environmental**	
Children at home	A [43]	Barriers	M [55]	Exercise frequency	M [51]	Having exercise partner	M [55]	Fitness center	M [52]
Drinking	A [45]	Behavioral beliefs	A [49]	Exercise limitations	M [56]	Having exercise role model	M [55]		
Gender	M [50]	Control beliefs	A [49]	Exertion during PA	A [43]	Providing feedback	A [47]		
Rural versus urban	A [47]/M [56]	Controllability	M [56]	General health	A [50]/M [50]	Promoting knowledge	A [47]		
ADT therapy	A [45]	Decision balance	A [48]	Physical functioning	M [50]	Promoting self-efficacy	A [47]		
Chemotherapy	M [56]	Expectations	M [55]			Support by friend/family	M [55]		
Chemotherapy day	A [43]	Expected success	M [51]			Support by health professional	A [47]		
Hormone treatment	A [46]/M [56]	Happiness	M [50]						
Lymphoma symptoms	A [46]/M [50]	Locus	M [51]						
Premenopausal	M [56]	Mood disturbance	A [42]						
Radiotherapy	M [56]	Negative affect	M [51]						
Relapse disease	A [41]/M [50]	Normative beliefs	A [49]						
Chemotherapy response	M [50]	PA enjoyment	M [55]						
Serological parameter	A [41]	PA fear	M [55]						
Time since diagnosis	M [50]	PA preference	M [50]						
Treatment status	M [50]	PA pros/cons	M [54]						
Treatment regime	M [50]	Perceived stress	A [46]						
Type of biopsy	A [43]	Perceived success	M [51]						
Type of surgery	M [52]	Personal control	M [51]						
		Planning	M [56]						
		Positive affect	M [51]						
		Self esteem	M [52]						
		Stability	M [51]						
		Symptoms	A [43]						
		Sleep disturbance	A [42]						
		Stage of change	M [54]						
		View on PA amount	A [43]						

A = determinants of exercise adherence; M = determinants of exercise maintenance.

ADT = Androgen Deprivation Therapy; PA = physical activity.

### Determinants of exercise maintenance

Six studies focused on determinants of exercise maintenance after completion of an intervention, and investigated 63 factors: 22 demographic and clinical, 28 psychosocial, nine physical, three social and one environmental factor. In total, nine demographic and clinical, ten psychological, and five physical factors were examined in two or more studies (Table 5). Due to inconsistent findings, there was insufficient evidence for an association of age, education, self-efficacy, instrumental and affective attitude, fatigue, quality of life, intention, PA intervention adherence, body mass index, baseline PA and cardiovascular fitness with exercise maintenance. Being married, income, employment, disease stage and social norms were examined in three studies, which all found no significant association with exercise maintenance (Table 5). There was insufficient evidence of 13 demographic and clinical, 18 psychological, four physical, three social and one environmental factor that were evaluated in one single study (Table 4).

**Table 5:** Determinants of exercise maintenance

	**N**	**N + (ref)**	**N- (ref)**	**N0 (ref)**	**LoE**
**Demographic & Clinical**					
Age	3		2 [52,56]	1 [53]	C
Education	3	1 [56]		2 [52,53]	C
Being married	3			3 [52,53,56]	C
Income	3			3 [52,53,56]	C
Employment	3			3 [52,53,56]	C
Smoking	2			2 [52,53]	C
Disease stage	3			3 [52,53,56]	C
Chemotherapy cycle	2			2 [52,53]	C
Type of chemotherapy	2			2 [52,53]	C
**Psychological**					
Self-efficacy	4	2 [54,56]		2 [53,55]	C
Instrumental attitude	3	2 [52,56]		1 [53]	C
Affective attitude	3	1 [56]		2 [52,53]	C
Fatigue	3		2 [52,56]	1 [53]	C
Quality of life	3	1 [56]		2 [52,53]	C
Intention	3	2 [53,56]		1 [52]	C
Perceived behavioral control	2			2 [52,53]	C
Social norms	3			3 [52,53,56]	C
Anxiety	2			2 [52,53]	C
Depression	2			2 [52,53]	C
**Physical**					
PA intervention adherence	4	2 [51,56]		2 [52,53]	C
Body mass index	4		2 [52,54]	2 [53,56]	C
PA at baseline	3	2 [52,56]		1 [53]	C
Body composition	2			2 [52,53]	C
Cardiovascular fitness	2	1 [54]		1 [50]	C

N+, number of studies showing a positive association; N-, number of studies showing a negative association; N0, number of studies showing no association.

LoE, Level of Evidence: A. strong evidence; B. moderate evidence; C. insufficient evidence.

PA = physical activity.

## Discussion

This study provides a comprehensive overview of determinants of exercise intervention adherence and exercise maintenance after completion of an intervention in cancer survivors. Eighteen studies were evaluated using a socio-ecological model of determinants of health behaviors, taking into account demographic and clinical, psychological, physical, social and environmental factors. Most studies examined demographic and clinical, psychological and physical factors, whereas few studies investigated social and environmental factors. We found moderate evidence for a positive association between exercise history and exercise adherence. For most demographic and clinical factors, we found insufficient evidence of an association with exercise adherence or maintenance. For exercise adherence, inconsistent findings were found for gender, type of treatment, as well as for psychological factors including perceived behavioral control, stage of change, self-efficacy, extraversion, the physical factor cardiovascular fitness and the environmental factor location of the fitness center. For exercise maintenance, we found inconsistent findings for age, education, self-efficacy, fatigue, attitude, quality of life, intention, PA intervention adherence, body mass index, baseline PA and cardiovascular fitness.

Similar to the review of Szymlek-Gay and colleagues [25], lower age, lower body mass index, more advanced disease stage, higher degree of readiness to change PA behavior, higher self-efficacy, higher physical fitness, and higher baseline PA were identified as possible determinants of exercise adherence. However, according to our best evidence synthesis, the level of evidence was insufficient mainly due to inconsistent findings across studies. In contrast to our review, Husebø et al. found exercise stage of change, intention, perceived behavioral control, and subjective norm to be a significant determinant of exercise adherence in their meta-analysis [23]. However, although statistically significant, the strength of the associations were low (<0.3). They extracted their results from univariate analysis instead of multivariate analysis which may have overestimated the strength of the associations.

Most demographic and clinical factors were not significantly associated with exercise adherence or maintenance. The lack of statistically significant associations may be related to small sample sizes and the relatively low variability of exercise adherence and maintenance. Most studies were conducted as efficacy trials, evaluating the effects of exercise in ideal circumstances, in which usually a more homogenous group of patients participated with a relatively high adherence [57]. On the contrary, effectiveness trials evaluating intervention effects under “real-world” conditions, generally have lower adherence levels [57]. More well-powered studies are needed on determinants of exercise adherence in real-world circumstances. Although, most demographic and clinical factors, such as age, gender and type of cancer, are unmodifiable, insight into these factors provide valuable information about which subgroups of patients that are more or less likely to adhere to exercise programs or maintain exercise behaviors.

From previous research, it is well known that social factors including social support, having an exercise partner or role model, may influence exercise behavior [55] or exercise behavior change [58]. From studies in the general population it is also known that the physical or built environment improving the availability, accessibility, and attractiveness of exercise opportunities (e.g., sidewalks, bicycle lanes, safe road crossings, availability of green spaces and recreation facilities) are related to exercise behavior [26]. Because cancer survivors may experience even more barriers than the general population, social support, as well as attractive and easily accessible exercise facilities may even be more important determinants for cancer survivors compared to the general population. However, only few studies have evaluated the association of social and environmental factors with exercise adherence and maintenance in cancer survivors. The few studies published to date suggest that feedback from trainers or nurses was positively associated with exercise adherence [47], whereas no significant association was found of social support, having an exercise partner or role model [55] and the location of the fitness center [52] with exercise maintenance. Future studies are needed to further build the evidence for the influence of social and environmental factors on exercise adherence and maintenance.

### Methodological quality

Overall, the methodological quality of the reviewed studies was low, with only one study of high quality [40]. A major concern regarding the quality of most included studies was the high likelihood of selection bias and small sample sizes. The included studies conducted secondary data analysis of RCTs that were not designed to evaluate determinants of exercise adherence. Further, many studies did not report point estimates and measures of variability. Another frequent methodological shortcoming was the lack of valid and reliable measures of adherence and maintenance. We recommend to systematically report session attendance in a supervised exercise intervention and/or using accelerometers of pedometers to assess PA levels.

### Strengths and limitations

Strengths of this systematic review include the extensive literature search in multiple relevant databases, the in-depth methodological quality assessment and best evidence synthesis, as well as the presentation of determinants within ecological framework categorizing demographic and clinical, psychological, physical, social and environmental factors. Another strength is the attempt to differentiate determinants of adherence to exercise interventions at different time points during cancer survivorship according to the PACC framework. However, due to the limited number of studies we were unable to study differences in determinants of exercise adherence before, during and after cancer treatment. The limited number of studies also hampered us to examine whether determinants of exercise adherence vary across cancer types and exercise modalities such as mode (e.g. aerobic versus resistance exercises), delivery (e.g. supervised versus home-based), intensity and frequency. Further work is necessary to determine the most important determinants of exercise adherence and maintenance, and to study differences across cancer types and exercise modalities. Another limitation is the variety of definitions of exercise adherence, with some studies exclusively focusing on adherence, whereas other studies also incorporated a measure on compliance, i.e. whether the PA was conducted at the prescribed intensity [22]. As a result, we could not differentiate between determinants of exercise adherence and determinants of compliance. Therefore, future studies should more clearly distinguish exercise adherence and compliance. Finally, similar to other reviews and meta-analysis, publication bias cannot be ruled out.

## Conclusion

This systematic review showed that exercise history was positively associated with exercise adherence. Further, inconsistent findings were found for age, gender and education as well as for psychological factors such as stage of change, attitude, intention, perceived behavioral control, self-efficacy, extraversion, fatigue, and quality of life, and physical factors including cardiovascular fitness, body mass index, and baseline PA. Future effectiveness trials are needed on the influence of social and environmental factors on exercise adherence and maintenance in addition to demographic, psychological and physical factors. In addition, future studies should provide insight into differences in determinants across timing of exercise interventions (e.g. before, during and after cancer treatment), cancer types and exercise modalities.

## Competing interests

The authors declare that they have no competing interests.

## Authors’ contribution

All authors have made substantial contributions to the conception and design of the study. CK, FJ and LB drafted, and WM, AM, JB, and MC critically revised the manuscript. All authors read and approved the final manuscript.
